# Intensive Care Nurses’ Pain Management Experiences within the Framework of the Biopsychosocial-Spiritual Model in Türkiye: A Qualitative Approach

**DOI:** 10.1007/s10943-025-02251-4

**Published:** 2025-01-30

**Authors:** Aylin Bilgin, Sinem Öcalan, Mustafa Sabri Kovancı

**Affiliations:** 1 Internal Medicine Nursing Department, Faculty of Health Sciences, Sakarya University of Applied Sciences, 54400 Sakarya, Turkey; 2 Psychiatric Nursing Department, Faculty of Nursing, Hacettepe University, 06100 Ankara, Turkey

**Keywords:** Biopsychosocial-spiritual model, Intensive care, Nurse, Pain, Symptom management

## Abstract

Pain, which includes biological, psychological, social and spiritual factors, is a common symptom experienced by patients in intensive care. This study aimed to uncover intensive care nurses’ perspectives on pain management strategies, employing the biopsychosocial-spiritual model as the guiding framework. This research employed a descriptive qualitative method, engaging participants from diverse locations across five provinces and eight different institutions. The study involved 16 intensive care nurses and utilized semi-structured online Zoom interviews. Data analysis was conducted using Braun and Clarke’s six stages, and reporting followed the consolidated criteria for qualitative studies. The answers of the nurses were grouped under four themes and six subthemes: (1) biological interventions, (2) psychological interventions, (3) social interventions: involving families in the process and (4) spiritual interventions: support religious activities. This study shows that intensive care nurses benefit from many practices in pain management. These interventions included medication management and ensuring physical comfort in the biological factor, distracting activities and being with the patient in the psychological factor, involving the family in care in the social factor and providing an environment that supports the patient’s religious needs under the spiritual factor.

## Introduction

Pain, a personal experience influenced by a combination of biological, psychological, spiritual and social factors, is frequently experienced by patients in intensive care units (ICU) (Malik, 2020; Olsen et al., 2015). Self-reporting of pain is widely regarded as considered the gold standard for assessing pain (von Baeyer, 2006). However, in the context of the ICU, patients frequently experience difficulties in expressing their pain due to the use of sedation (Devlin et al., 2018; Rose et al., 2011). This may cause their pain under-recognition and inadequate management of their pain (Hylén et al., 2020).

Patients discharged from the ICU often report experiencing pain, even though they were unable to express it during their hospitalization (Hylén et al., 2020). In a study, patients stated that they tried to express their pain because they were intubated or sedated, but they felt lonely and helpless because they could not express it (Hylén et al., 2020). Inadequate pain management in intensive care can cause anxiety, sleep deprivation, fatigue delirium, and agitation in patients (Chanques et al., 2006). In addition, ensuring pain management allows patients to be weaned off mechanical ventilation in a short time and shortens the length of stay in hospital and intensive care (Bhattacharyya et al., 2023).

Nurses are among the key professionals who closely observe and evaluate this process, often taking a leading role in providing pain management (Song, et al., 2015). However, studies have shown that intensive care nurses lack knowledge in pain assessment and that some of them do not use pain assessment tools for patients who are unable to communicate (Siddiqui et al., 2023; Youinis, 2021). Critical care nurses cited the patient’s inability to communicate, patient instability and lack of protocols/guidelines for pain assessment as the most common barriers to evaluation and management of pain (Rababa et al., 2023).

### Background

A wide range of methods are used by nurses to manage pain, from pharmacological interventions to non-pharmacological approaches. Although pharmacological interventions are the focus of the literature for pain management in the ICU, psychological methods such as mental imagery and distraction and physical methods such as positioning, acupuncture and touching are also used (Harvin & Kao, 2020; Ismail et al., 2019). Furthermore, nurses have reported that non-pharmacological methods such as listening to music listening, massage and providing physical comfort are as effective as medication in pain management of patients (Bhattacharyya et al., 2023; Golino et al., 2023). Additionally, studies based on the biopsychosocial-spiritual model emphasize that pain has biological, psychological, sociological and spiritual factors (Matteliano et al., 2014; Taylor et al., 2013).

This model provides a framework for managing disease-related symptoms and identifying individuals’ coping resources (Engel, 1977; Lysne & Wachholtz, 2010). In the literature, studies based on the biopsychosocial-spiritual model have focused on biological, psychological, sociological and spiritual factors that cause pain (Matteliano et al., 2014; Taylor et al., 2013). No study presents the methods nurses use in intensive care pain management based on this model. This study aims to reveal the experiences of intensive care nurses regarding the methods they use in pain management based on the biopsychosocial-spiritual model.

### Theoretical Framework

The Biopsychosocial-Spiritual Model, an extension of George Engel’s, 1977 Biopsychosocial Model, provides a holistic alternative to the traditional biomedical model and served as the framework for this study. Engel’s original model proposed that health and disease encompass biological, psychological and social dimensions (Engel, 1977). With growing evidence supporting the influence of religion and spirituality on health behaviors, coping and quality of life, spirituality was later incorporated into the model, resulting in its revision as the biopsychosocial-spiritual model (Sulmasy, 2002). The biopsychosocial-spiritual model is widely used in pain research, offering a comprehensive approach to understanding and managing pain (Matteliano et al., 2014; Taylor et al., 2013).

In this model, although the contents of biological, psychological, social and spiritual factors are different, they are parts of the whole (Table 1). Each factor may be affected differently by the individual’s history and disease and may interact with and influence the individual’s other factors (Engel, 1977; Sulmasy, 2002). Therefore, it is recommended to use the biopsychosocial-spiritual model in pain management, as it covers the individual entirely and provides evaluation (Taylor et al., 2013).

**Table 1:** Integration of the study into biopsychosocial-spiritual model dimensions

Biopsychosocial-spiritual model dimensions	Description of dimensions	Semi-structured questionnaire	
Biological dimension	This dimension includes cells, neurotransmitters, drugs used for the disease, physical disability and posture (Engel, 1977; Mescouto et al., 2022)	Question 1	What are the biological/pharmacological methods you use for pain management? Probe: What effect do these methods have on pain? Probe: What difficulties do you encounter when using these methods? Probe: In what ways do these methods need to be improved? Probe: What kind of feedback did you receive from patients after using these methods?
Psychological dimension	This dimension includes such as coping skills, thoughts, attitudes and mental health (Engel, 1977; Mescouto et al., 2022)	Question 2	What are the psychological methods you use for pain management? Probe: What effect do these methods have on pain? Probe: What difficulties do you encounter when using these methods? Probe: In what ways do these methods need to be improved? Probe: What kind of feedback did you receive from patients after using these methods?
Social dimension	This dimension includes interpersonal relationships, work life, family life and socioeconomic status (Engel, 1977; Mescouto et al., 2022)	Question 3	What are the social methods you use for pain management? Probe: What effect do these methods have on pain? Probe: What difficulties do you encounter when using these methods? Probe: In what ways do these methods need to be improved? Probe: What kind of feedback did you receive from patients after using these methods?
Spiritual dimension	This dimension includes the meaning and purpose of life, religion and belief (Saad et al., 2017)	Question 4	What are the spiritual methods you use for pain management? Probe: What effect do these methods have on pain? Probe: What difficulties do you encounter when using these methods? Probe: In what ways do these methods need to be improved? Probe: What kind of feedback did you receive from patients after using these methods?

## Methods

### Study Design

The research was conducted with a descriptive qualitative method. This method aims to gain an in-depth understanding of a specific issue or situation by examining and analyzing it in detail (Doyle et al., 2020). This study was reported according to consolidated criteria for qualitative studies (COREQ) (Tong et al., 2007).

## Research Team and Reflexivity

The researcher (Aylin Bilgin) has a PhD degree in internal medicine nursing and works as an assistant professor at the faculty of nursing of a university. The researcher (Sinem Ocalan) is a PhD degree in psychiatric nursing and works as research assistants in the psychiatric nursing department of the same university. Finally, the researcher (Mustafa Sabri Kovanci) is doctoral students in psychiatric nursing and work as research assistants in the psychiatric nursing department of the same university. All researchers have qualitative research experience.

### Setting and Sample

Purposive sampling was used as the sampling method. The first participant was determined through purposive sampling, a method where individuals are selected based on specific characteristics that align with the study’s research objectives. Purposive sampling is based on the selection of individuals, situations or events that can provide the most appropriate information for the purpose of the research according to consciousness and characteristics (Campbell et al., 2020). The inclusion criteria for the study were intensive care nurses who (a) actively worked in the adult ICU, (b) provided care to at least one patient who verbally reported their pain, (c) could be reached via online platforms (WhatsApp®, Facebook®, Instagram® and email) and (d) volunteered to participate in the study. The exclusion criteria for the study were intensive care nurses who (a) worked in a pediatric or neonatal ICU and (b) lacked Internet access.

The researchers used online platforms (WhatsApp®, Facebook®, Instagram® and email) to announce the study to interested participants. Intensive care nurses who met the inclusion criteria and volunteered to participate in the study were contacted verbally (by phone) or in writing (via text and email). Interviews were conducted through Zoom®. Accordingly, 17 intensive care nurses reached out to the researchers to participate in the study, but one was excluded from the study because she worked in a pediatric intensive care clinic. The study was completed with 16 intensive care nurses.

### Data Collection

The research data were collected through online Zoom interviews using a semi-structured questionnaire between July 2023 and January 2024. The semi-structured questionnaire was developed in line with the Biopsychosocial-Spiritual Model. The researchers used semi-structured questions to elicit the experiences of intensive care nurses toward the factors of the biopsychosocial-spiritual model.

The components of the biopsychosocial-spiritual model were applied to all questions in this form: Question 1 concerns biological interventions, question 2 psychological interventions, question 3 social interventions and question 4 spiritual interventions. To assess the applicability of the semi-structured questionnaire, a pilot interview was conducted with two participants who met the inclusion criteria. The participants in the pilot study were included in the sample of the study since the research questions were not revised.

Before starting the interviews, detailed information about the purpose and methodology of the study was provided. In-depth interviews were conducted until no new information about the phenomenon was obtained. Data saturation was judged to have been reached when no new information emerged. After the thirteenth interview, the researchers did not observe any new data on pain management of intensive care nurses. However, three more verification interviews were conducted to confirm that no new data emerged (Polit & Beck, 2021). The interviews were conducted via Zoom and lasted between 25 and 45 min (average 32 min).

### Data Analysis

The MAXQDA 18 software was utilized to organize the themes and subthemes systematically. Braun and Clarke’s (2022) six-stage ((a) familiarization with data; (b) initial code generation; (c) theme search based on initial codes; (d) theme review; (e) theme definition and labeling; and (f) report writing) thematic analysis approach was used to provide a comprehensive framework for analyzing the data (Braun & Clarke, 2022). The interviews were analyzed repeatedly by each researcher independently. Codes, themes and subthemes were discussed by the researchers and determined by consensus. The dimensions of the Biopsychosocial-Spiritual Model, which constitute the theoretical foundations of the study, were organized as the themes of the current study, and subthemes related to these themes were determined and presented with a systematic evaluation.

### Rigor

The rigor of this study was ensured through the strategies suggested by Lincoln and Guba (Lincoln & Guba, 1985). To improve the credibility of the findings, a methodological approach was adopted in the data analysis phase, and this process was explained in detail. The fact that the data were analyzed and interpreted by more than one researcher also reinforced reliability. In addition, participant triangulation was ensured by including participants from 5 provinces and 8 different institutions. Thus, multidimensional information on the pain management experiences of intensive care nurses was obtained.

Confirmability is critical to ensure the objectivity of the research. All interviews were conducted consistently using the same interview form. The fact that the research process and results were evaluated by three researchers experienced in qualitative research supports the confirmability and dependability of the study. In addition, the interview data were constantly compared and reviewed between the researchers and finalized by consensus. All processes of the study such as sampling method, data collection procedures and analysis methods are presented in detail in the article to ensure the transferability of the study.

### Ethical Considerations

Ethical approval to conduct the study was obtained from the Sakarya University of Applied Sciences Ethics Committee (approval dated 21.02.2023 and numbered 28/7). The principles of the Declaration of Helsinki were followed in this study. Both written and verbal informed consent was obtained from all participants.

## Results

**Fig. 1:**
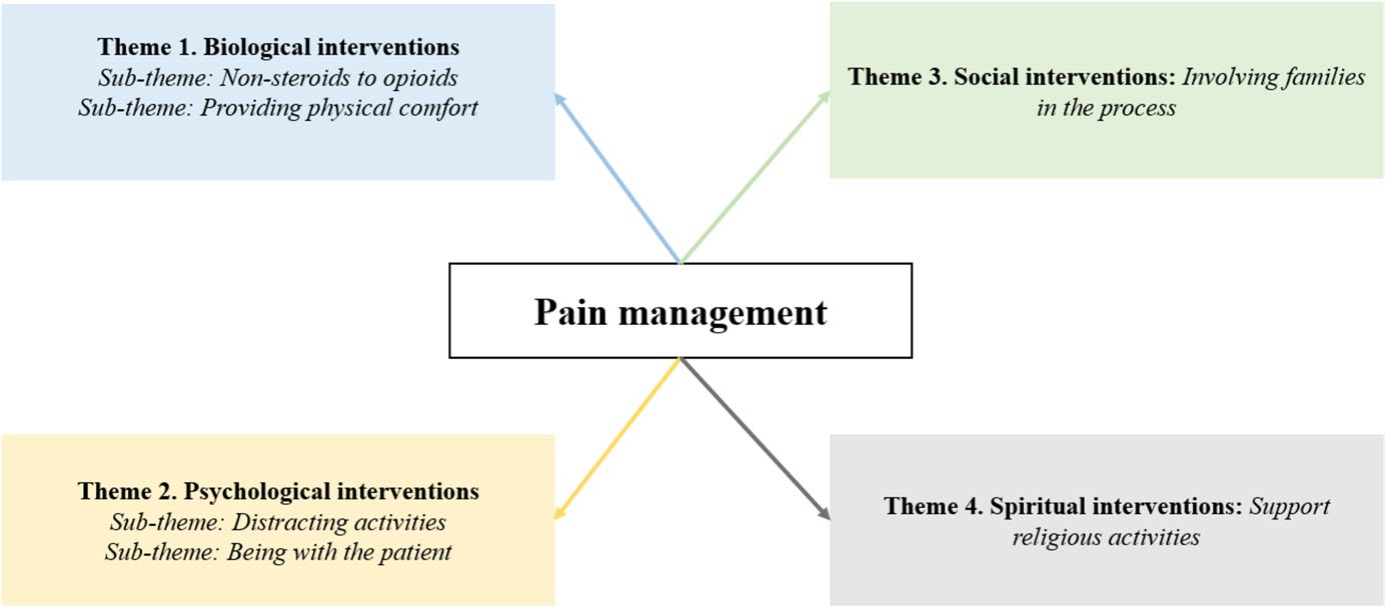
The main themes and subthemes of pain management

The majority of the participants was women (93.75%) and had undergraduate degrees (75%). The ages of the participants ranged from 24 to 33 years. Participants had experience working in ICU ranging from three months to five years. The majority of patients did not receive any training on pain management and used pharmacology in pain management (Table 2). Figure 1 presents the main themes and subthemes regarding pain management.

**Table 2:** Descriptive characteristics of participants (*n* = 16)

	Age	Gender	Marital status	Education level	Duration of professional experience	Working time in intensive care	Intensive care department	Status of receiving training on pain management	The most used method in pain management
N1	25	Female	single	undergraduate	1 year	1 year	Internal medicine	No	Pharmacological
N2	30	Female	married	undergraduate	5 years	4 years	General surgery	No	Pharmacological
N3	25	Female	single	undergraduate	1 year	1 year	Internal medicine	No	Pharmacological
N4	28	Female	married	undergraduate	3.5 years	3 years	Brain surgeon	No	Pharmacological
N5	26	Female	single	postgraduate	2 years	2 years	Chest diseases	Yes	Pharmacological
N6	27	Female	single	undergraduate	3 years	3 years	Brain surgeon	No	Pharmacological
N7	31	Female	married	undergraduate	6 years	5 years	Chest diseases	No	Pharmacological
N8	33	Female	married	undergraduate	8 years	4 years	Chest diseases	No	Pharmacological
N9	24	Female	single	undergraduate	1 year	1 year	Brain surgeon	Yes	Pharmacological
N10	34	Female	married	undergraduate	17 years	5 years	Palliative	No	Pharmacological, psychological
N11	28	Female	single	postgraduate	4 years	4 years	Internal medicine	No	Pharmacological
N12	24	Female	single	undergraduate	1.5 years	1.5 years	Internal medicine	No	Pharmacological
N13	25	Female	single	postgraduate	2 years	1.5 years	Emergency critical care	No	Pharmacological, psychological
N14	24	Female	single	undergraduate	1 year	1 year	Anesthesia	Yes	Pharmacological
N15	29	Male	married	postgraduate	6 years	1 year	Internal medicine	No	Pharmacological, psychological
N16	25	Female	married	undergraduate	1.5 years	1 year	Internal medicine	No	Pharmacological

### Theme 1. Biological Interventions

#### Subtheme: Nonsteroids to Opioids

Nurses stated that they mostly applied pharmacologic interventions to patients experiencing pain in the ICU. Pharmacologic methods are applied in accordance with the doctor’s order from weakly effective to strongly effective depending on the severity of the disease and the location of the pain.

‘‘I mostly use pharmacological methods to manage pain, starting with nonsteroid medications according to the doctor’s order and progressing to narcotic analgesics depending on the patient’s condition’’ (N15)*.*

“Depending on the severity of the pain or the location of the pain, I try to alleviate the pain by using pharmacological methods.” (N13)

#### Subtheme: Providing Physical Comfort

Nurses stated that pain is sometimes caused by the position or the medical devices used, such as oxygen devices. Therefore, nurses emphasized that changing the position, massage or the use of protective barriers appropriate to the devices used is an effective method to reduce pain.

“Especially if the patient has lower extremity pain, I do a massage. I massaged the patient’s leg with a little bit of Vaseline. They usually relax after that.” (N12)

“In patients who lie down for a long time or in patients with pressure injuries, pain can be caused by position. For this reason, I position patients frequently and support them with pillows.” (N3).

“Oxygen masks cause pain because they apply pressure to the face, and I try to alleviate mask-related pain with barrier preventions.” (N7)

### Theme 2. Psychological Interventions

#### Subtheme: Distracting Activities

The nurses emphasized that they tried to draw the patient’s attention in a different direction to make them forget the pain. They especially tried to distract them from the feeling of pain by talking about daily life and family. They also used distraction methods such as turning on the television, playing music and focusing on breathing.

“I try to play music to the patient using the radio or Walkman. I try to play music that the patient likes.” (N11)

“I use the deep breathing method. I try to calm them down by breathing deeply.” (N10)

“If I can, I turn on the television to distract the patient.” (N12)

“I talk to the patients about family, and different topics from daily life. The patient’s talking to me without focusing on the pain at least alleviates the pain for a while and makes them forget.” (N9).

#### Subtheme: Being with the Patient

Nurses stated that uncertainty and anxiety about the pain process in patients made pain management difficult. For this reason, nurses indicated that interacting with patients, touching the patient and informing about the pain process have an important role in pain management.

“I hold the patient’s hand. I try to make contact with the patient as much as possible. It’s good. The patient takes my hand in return, and we talk.” (N14)

“The intensive care environment is stressful. The patient may feel pain due to stress. Telling the patient how long the pain may continue, that we understand them, and that we will intervene helps the patient to calm down.” (N1).

### Theme 3. Social Interventions: Involving Families in the Process

Nurses reported that patients’ interaction with their families alleviated pain. In particular, patients were encouraged to bring a phone with them, as it is not always possible to be in contact with family in intensive care. In addition, nurses allowed additional visiting time for patients whose pain persists so that they could spend more time with their relatives.

“Sometimes my patients say that their pain is relieved when they see their family. For this reason, it is important to give them a phone to stay in continuous contact.” (N15)

“We ensure that the patient’s relatives feed the patient who does not want to eat due to pain, allowing them to spend more time with their family.” (N16)

“We invite a member of the patient’s family to spend the night with them. Because sometimes, no matter what we do, the pain does not go away. But patients forget when their relatives are with them.” (N6).

### Theme 4. Spiritual Interventions: Support Religious Activities

All the nurses who participated in this study identified as members of the Islamic faith. Additionally, they reported that the majority of the patients they cared for were also of the Islamic faith. Nurses who recognized that spiritual care is not limited to religious components stated that they contributed to alleviating the pain of patients in intensive care by solely supporting the religious practices that the patients and their families wished to perform. Patients who wanted to pray to God by their beliefs were supported. In addition, patients’ relatives who wanted to pray or read the Quran to their patients were given time.

“We have patients who want to hear the adhan, and who want to pray. They want to do ablution. We prepare an environment so that patients feel comfortable. Otherwise, patients become aggressive and angry.” (N16).

“I try to support the patient’s prayer and communication with God. I also support them by remaining silent, allowing them to pray, and leaving them alone.” (N9)

“Time and environment are provided for relatives of patients to come in and pray or read the Quran” (N5)

## Discussion

This study confirmed that pain management is a multidimensional concept and showed that nurses utilize biological, psychological, social and spiritual methods in pain management in intensive care. In particular, nurses use pharmacologic methods effectively and also utilize practices such as diverting attention and family support.

Pharmacological interventions are known to be widely utilized in clinical settings that frequently encounter patients with pain, such as intensive care units (Shaikh et al., 2018). In this study, participants reported frequent use of pharmacological agents, ranging from less effective to very effective, while providing pain control in the ICU. In the literature, many studies have extensively highlighted the efficacy of pharmacological applications for pain and demonstrated their analgesic effects (Verret et al., 2020; Yang et al., 2019). It is recommended to start with nonsteroidal pain relievers, taking into account the individual’s response. If the pain persists, it is recommended to progress to opioids (Small & Laycock, 2020). While physicians are responsible for prescribing pharmacological treatments, nurses play a critical role in administering these medications and managing potential adverse effects (Piyakhachornrot & Youngcharoen, 2024).

Another notable finding of the study was that participants managed pain in patients through non-pharmacological practices such as massage and repositioning. Pados and McGlothen-bell (2019) found that massage specifically reduced pain intensity. Another study suggested passive movement and position change in intensive care patients reduced pain (Tanvir et al., 2023). Additionally, cold and hot compresses, reflexology and aromatherapy have been shown to be effective in pain management (Fan & Chen, 2020; Mota et al., 2019). Non-pharmacological practices are particularly valuable as they can be performed independently by nurses without risk of side effects.

Another key finding of the study is that participants observed a reduction in pain by using distraction techniques in pain management and by spending time with the patient and discussing their anxiety. Studies have found that music therapy effectively reduces pain in some individuals (Richard-Lalonde et al., 2020; Yarahmadi et al., 2018). In addition, providing emotional support to patients in intensive care (Martorella, 2019), performing distracting activities (Sandvik et al., 2020) and using cognitive behavioral techniques (Burns et al., 2022) have also been found effective for pain management. The increased use of these non-pharmacological methods could facilitate more effective pain management while reducing the need for unnecessary pharmacological interventions.

Nurses in this study highlighted the importance of family support in pain management, noting that it contributed to alleviating patients’ pain. They also reported utilizing patient visits to facilitate family support. Studies have shown that social support practices are an effective way to reduce pain (Matthias et al., 2022; Munkombwe et al., 2020). One study reported that conversations and physical touch from family members reduced patients’ pain complaints, emphasizing the significant role of the family in pain management (Munkombwe et al., 2020). Sturgeon et al. (2016) concluded that social support and satisfaction with social relationships are an important determinant of pain in patients (Sturgeon et al., 2016). A systematic review emphasized that social support-based practices applied to individuals with pain are effective in alleviating pain (Che et al., 2018). Social support practices may be effective in alleviating pain because they facilitate patients’ coping with pain, draw their attention to a different direction and contribute positively to the patient’s acceptance of the disease.

Another important finding of this study was the use of spirituality in pain management, with nurses creating time and space for patients and their relatives to pray. Spirituality, a critical component of holistic care, significantly influences the perception and management of illness-related pain (Siler et al., 2019). Research has revealed a relationship between spiritual distress and pain (Harris et al., 2018). Furthermore, individuals experiencing pain have reported spiritual needs, such as praying or participating in religious ceremonies, and have expressed a desire for healthcare professionals to address these needs (Büssing et al., 2013; Hasenfratz et al., 2021). Spiritual care can help individuals improve their pain coping skills (Aydın Yıldırım & Kes, 2022). Accordingly, in this study, nurses’ attention to patients’ spiritual needs and their efforts to address these needs within the constraints of the ICU environment likely contributed positively to pain management.

### Strengths and Limitations

The strengths of the study lie in the use of the Biopsychosocial-Spiritual Model, which offers a holistic approach to understanding pain management in ICU. The qualitative design provided rich and in-depth insights into the nurses’ experiences. The study, with a diverse participant pool, offers valuable practical outcomes for improving comprehensive pain management in critical care settings. There are three main limitations of this study. The first is that the interviews were structured online and therefore nurses who could not access the online platform did not have the opportunity to participate. The second is that the majority of the participants was female nurses. The third limitation is the sample size; the study results can be confirmed with a larger sample.

### Implications for Practice

Addressing all factors of pain (biological, psychological, social and spiritual) can improve the quality of care by managing it in a short time. For this, it is necessary to first evaluate the pain with short inventories and learn the individual’s coping skills and needs in pain management. Short questions can be added to nurses’ data collection forms regarding these skills and needs, and in this way, individuals’ needs can be prevented from being ignored. In addition, nurses can be taught biological, psychological, social and spiritual methods for pain management by organizing in-house training and seminars.

## Conclusion

Pain is an experience that includes biological, psychological, spiritual and social factors. Adopting a holistic evaluation and approach that can address all factors in pain management can facilitate the management of the process. Intensive care nurses frequently encounter patients experiencing pain and manage the process. In this study, the interventions they implemented for all factors of pain were presented. The biological factor includes medication management and ensuring physical comfort, the psychological factor includes using distracting activities and being with the patient, the social factor includes involving the family in care and the spiritual factor includes providing an environment where the patient can meet their religious needs.
